# Epidemiological and molecular features of dengue virus type-1 in New Caledonia, South Pacific, 2001–2013

**DOI:** 10.1186/1743-422X-11-61

**Published:** 2014-03-31

**Authors:** Myrielle Dupont-Rouzeyrol, Maïté Aubry, Olivia O’Connor, Claudine Roche, Ann-Claire Gourinat, Aurélie Guigon, Alyssa Pyke, Jean-Paul Grangeon, Eric Nilles, Suzanne Chanteau, John Aaskov, Van-Mai Cao-Lormeau

**Affiliations:** 1 URE- Dengue et autres Arboviroses, Institut Pasteur de Nouvelle-Calédonie, Réseau International des Instituts Pasteur, 98800 Nouméa, Nouvelle-Calédonie; 2 Institut Louis Malardé, 98713 Papeete Tahiti, Polynésie Française; 3 Institut Pasteur de Nouvelle-Calédonie, Réseau International des Instituts Pasteur, 98800 Nouméa, Nouvelle-Calédonie; 4 Queensland Health Forensic and Scientific Services, QLD 4108 Coopers Plains, Australia; 5 Direction des Affaires Sanitaires et Sociales, 98800 Nouméa, Nouvelle-Calédonie; 6 Pacific Technical Support Division, World Health Organization, PO BOX 113, Suva, Fiji; 7 Queensland University of Technology, Brisbane QLD 4001, Australia

**Keywords:** Dengue, Phylogeny, Genotype, Epidemics, New Caledonia

## Abstract

**Background:**

The epidemiology of dengue in the South Pacific has been characterized by transmission of a single dominant serotype for 3–5 years, with subsequent replacement by another serotype. From 2001 to 2008 only DENV-1 was reported in the Pacific. In 2008, DENV-4 emerged and quickly displaced DENV-1 in the Pacific, except in New Caledonia (NC) where DENV-1 and DENV-4 co-circulated in 2008–2009. During 2012–2013, another DENV-1 outbreak occurred in NC, the third DENV-1 outbreak in a decade. Given that dengue is a serotype-specific immunizing infection, the recurrent outbreaks of a single serotype within a 10-year period was unexpected.

**Findings:**

This study aimed to inform this phenomenon by examining the phylogenetic characteristics of the DENV-1 viruses in NC and other Pacific islands between 2001 and 2013. As a result, we have demonstrated that NC experienced introductions of viruses from both the Pacific (genotype IV) and South-east Asia (genotype I). Moreover, whereas genotype IV and I were co-circulating at the beginning of 2012, we observed that from the second half of 2012, i.e. during the major DENV-1 outbreak, all analyzed viruses were genotype I suggesting that a genotype switch occurred.

**Conclusions:**

Repeated outbreaks of the same dengue serotype, as observed in NC, is uncommon in the Pacific islands. Why the earlier DENV-1 outbreaks did not induce sufficient herd immunity is unclear, and likely multifactorial, but the robust vector control program may have played a role by limiting transmission and thus maintaining a large susceptible pool in the population.

## Findings

Dengue is the most prevalent arthropod-borne viral infection of humans in tropical and subtropical countries. Every year, dengue virus (DENV) infections cause more than 50 million cases, 500 000 hospitalizations and 12 500 deaths in the world [1,2]. DENV is a single-stranded, positive-sense RNA virus of the genus *Flavivirus* transmitted by *Aedes* mosquitoes. There are four distinct serotypes (DENV-1 to DENV-4) and infection with one does not provide long-term cross-protective immunity against the three others [3,4]. Based on the sequence of the envelope gene (E), each serotype may be divided into distinct genotypes often associated with specific geographical regions. Both epidemiological observations and *in vitro* studies suggest that distinct genotypes have different potential to cause severe dengue epidemics [5-7]. Thus, molecular epidemiologic studies have become a critical issue for understanding epidemic patterns of viral spread.

In the Pacific, dengue epidemics were mainly caused by a single serotype/genotype introduced from a hyper-endemic continental country. In individual Pacific Islands Countries and Territories (PICTs) the epidemiology of dengue is heterogeneous. Small PICTs sustain DENV transmission for only several months, while larger ones, like French Polynesia (PF) or New Caledonia (NC), may experience active circulation of a single serotype/genotype for several years until the emergence of a new epidemic viral strain [8-10]. In this study, we reconstruct the epidemiological and phylogenetic history of DENV-1 in NC during the last decade to better understand the NC dengue epidemiology.

**Figure 1:**
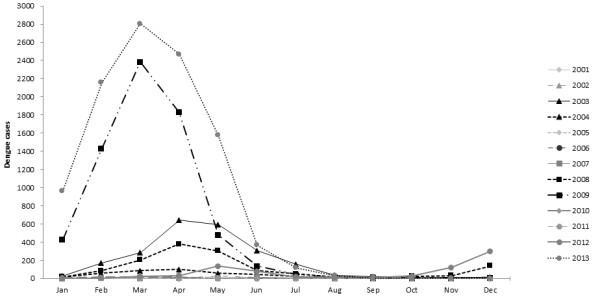
**Dengue epidemic profile in New Caledonia from 2001 to 2013.** The number of dengue cases represents the confirmed and probable cases. This Figure presents the succession of epidemic years (2003–2004, 2008–2009 and 2012–2013) and non-epidemic years in NC. It also highlights the seasonality of DENV epidemics in NC (peak in March or April).

In NC dengue epidemics have a 3–4 years cyclical pattern of occurrence [11] with dengue outbreaks usually lasting two years, with two peaks occurring during the two consecutive hot-and-rainy seasons and few cases reported during the cool season in-between (Figure 1). In 2001, as DENV-1 was emerging in several PICTs [9,11], imported dengue cases from PF were reported in NC. The following year, about 100 DENV-1 local cases were detected, announcing the large DENV-1 outbreak that hit the territory in 2003–2004 (Table 1) [11-13]. In 2007, after two years without any report of locally acquired DENV infections, DENV-1 cases were reported (Table 1). Half of them were imported cases from PF where a DENV-1 epidemic was still ongoing [14]. In November 2008, while DENV-1 transmission was still active, DENV-4 was detected in people coming back from Vanuatu where the serotype had recently emerged [10,15,16]. These were the first DENV-4 cases reported in NC for more than 25 years [17]. Despite an active surveillance, NC health authorities did not reach to prevent and control the introduction of DENV-4. In 2009, NC experienced an epidemic concomitantly involving DENV-1 and DENV-4 (Table 1) [13]. In 2010, no more DENV-4 cases were reported while DENV-1 continued to be detected until the end of year. The serotype switch observed in other PICTs did not occur in NC [10,16]. In 2011, no dengue cases were detected although the number of dengue suspected cases had dramatically increased due to the occurrence of the first chikungunya epidemic ever reported in NC [18,19]. Unexpectedly, DENV-1 re-emerged in 2012–2013 in NC and caused the largest outbreak ever reported (Figure 1 and Table 1) [13]. This outbreak was particularly severe: rapid increase of the number of cases, high rate of hospitalization in a short time, important transmission during the cool season, restart of active transmission early after the cool season.

**Table 1:** New Caledonia dengue epidemiological data from the years 2001 to 2013

**Year**	**Diagnostic demands**	**Confirmed cases**	**Probable cases**	**Imported cases**^**a,b**^	**Main DENV serotype**	**Number of deaths**^**b**^
2001	956	21	0	21	1	0
2002	1111	64	33	10	1	0
2003	7758	601	1997	nd	1	17
2004	2563	179	281	nd	1	2
2005	760	2	43	2	1	0
2006	904	9	44	8	1	0
2007	1012	33	6	19	1	0
2008	5262	1008	123	12	1 and 4	2
2009	14927	6328	968	13	1 and 4	3
2010	1841	25	67	1	1	0
2011	3567	2	10	2	-	0
2012	3986	654	54	3	1	1
2013	22375	8545	1380	33	1	4

The number of tests requests, confirmed cases (RT-PCR and/or NS1), probable cases (IgM only) and deaths probably linked to dengue are reported.

^a^Imported cases among the confirmed cases, ^b^Recorded by the Health Department of New Caledonia, *nd* not determined.

**Figure 2:**
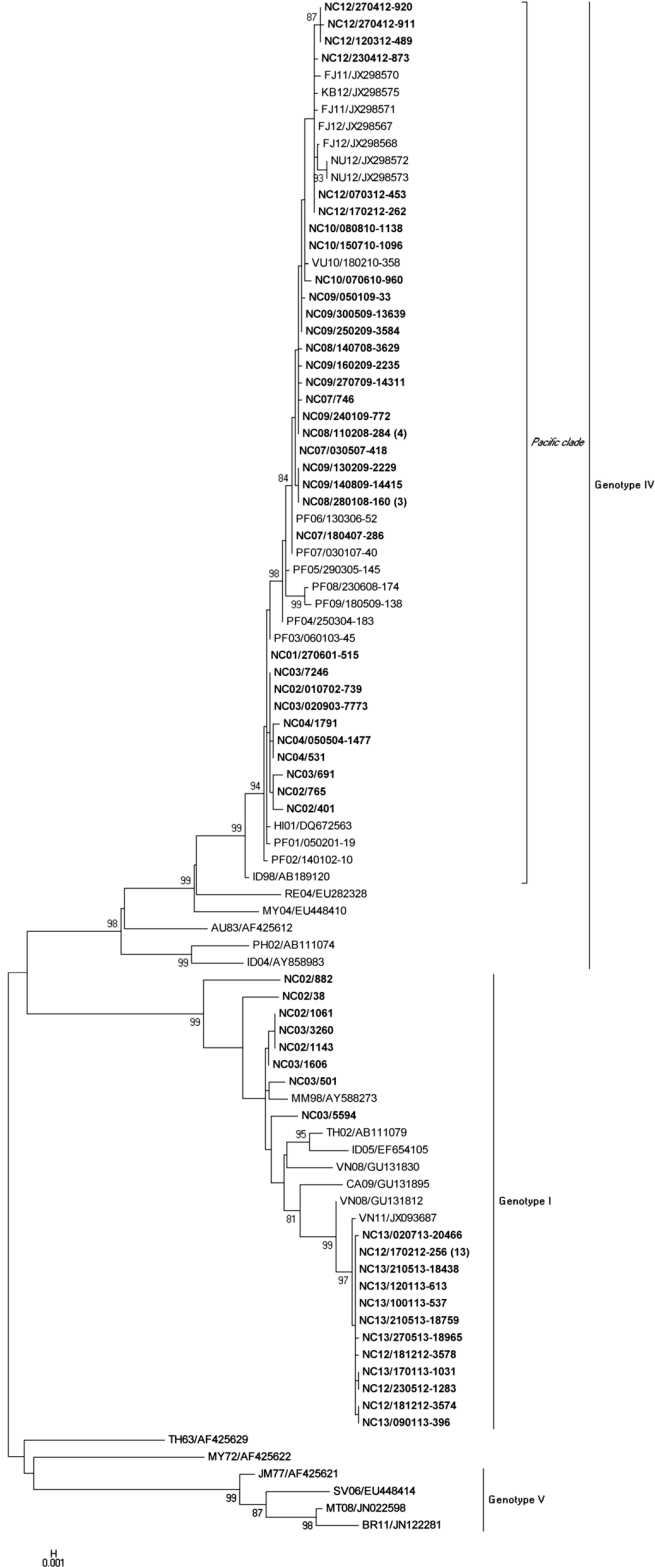
**Evolutionary relationships of E gene sequences of DENV-1 (1478 nt).** Maximum-Likelihood original trees derived from 110 DENV-1 E gene sequences (90 PICTs and 20 retrieved from GenBank). The percentage of replicate trees in which the associated taxa clustered together in the bootstrap test (1000 replicates) is shown for values over 80. The period (years) these strains have been collected is indicated just after the country isocode. The number in brackets represents the number of NC DENV-1 strains identical (nucleotide sequence) to the strain reported on the tree. The 73 (53 + 20 in brackets) NC DENV-1 strains are shown in bold. Genbank accession numbers of the DENV-1 E gene sequenced for this study are reported in Additional file 1.

In this study, envelope (E) genes from 90 DENV-1 viruses recovered in NC and other PICTs (Additional file 1) were amplified by RT-PCR and sequenced as described previously [16]. As DENV-1 is divided in five genotypes [5], manually designed oligonucleotide primers D1M/869F (5’-*GGAACATCCATCACCCAGAA*-3’) and D1NS1/2510R (5’-*CTCTGTCCAGGTGTGAACTT*-3’) were used for both amplification and sequencing of genotype IV viruses, additional primers D1E/1352F (5’-*GTGATCGTTACTGTCCACAC*-3’) and D1E/OOCF (5’- *GTAATAGTCACCGTCCACAC*-3’) were used to sequence genotype IV and I viruses respectively. Overlapping fragments were assembled with Staden Package (MRC Cambridge, England). Nucleotide sequences were aligned with the multiple sequence alignment software Clustal W integrated in MEGA version 5.0 software. Phylogenetic analysis was then carried out with MEGA5, using the Maximum likelihood method. The evolutionary distances were computed using the Kimura 2-parameter method defined as one of the best evolution model with MEGA5. As shown by the Figure 2, NC strains collected from 2001 to 2013 belong to genotypes I and IV. All sequenced genotype IV viruses recovered in NC during the last decade group together with other DENV-1 strains collected in PICTs during the same period within the “Pacific clade” previously described [9]. DENV-1/genotype IV viruses recovered in NC from 2002 to 2004 and from 2007 to 2012 are close to strains isolated in PF in 2001–2002 and in 2006–2007 respectively. This observation corroborates epidemiological data that suggested that the first two dengue outbreaks of the decade in NC resulted from introduction of DENV-1 viruses from PF. Interestingly, NC genotype IV viruses recovered in 2012 are closely related to DENV-1 strains collected in Fiji, Kiribati and Niue within the same year. Based on our findings, the re-emergence of DENV-1 genotype IV in NC in 2012 may have resulted from introductions of viruses from other PICTs. Genotype I viruses recovered in NC form two clearly independent clusters, the first cluster includes strains collected in 2002–2003 as mentioned before [8,20], the second cluster contains viruses isolated in NC in 2012–2013. This later cluster is close to a strain recovered in Vietnam in 2011 (VN11/JXD93687) what suggests that the re-emergence of DENV-1 genotype I in NC in 2012 resulted from a new introduction of a virus from South-east Asia. Moreover, the amino acids analysis showed that all NC 2012–2013 DENV-1/genotype I strains share 100% homology with the VN11/JXD93687 and VN08/GU131812 strains (data not shown). From February to July 2012, DENV-1 viruses recovered in NC belonged to either genotype I or IV. From August 2012 to the end of 2013, all the NC DENV-1 strains sequenced were genotype I (Figure 2). Moreover all DENV-1 positive sera collected in August 2012 and in August 2013 were all amplified by genotype I specific RT-PCR using the primers D1E/MDRF (5’- *GAAATATTCAGTAATAGTCACCGT*-3’) and D1EREV-R (5’- *CATGGTGCATCTGTTCC*-3’) (data not shown). These data are suggesting that a switch occurred in the genotype circulating as observed in hyperendemic countries [21].

The occurrence of two consecutive outbreaks due to the same dengue serotype had historically already been observed in NC and other PICTs [7,11]. Thus, the re-emergence of DENV-1 in NC in 2008, i.e. five years after the previous DENV-1 outbreak, was not surprising, particularly given the active circulation of the virus in PF. However, what was unexpected was the fact that DENV-1 was not replaced by DENV-4 during the 2008–2009 outbreak and that both serotype circulated during more than a year. The absence of serotype replacement contrasts with the historical data on the epidemiology of dengue in PICTs and with the situation reported during the same period in other PICTs [10,15]. Otherwise in contrast with the NC historical dengue epidemiological profile [11] DENV-1 re-emerged in NC in 2012 although this serotype had already circulated in the country for ten years. As suggested by our phylogenetic analyses the recurrent re-emergences of DENV-1 over the last decade in NC mainly resulted from introductions of “Pacific clade” genotype IV viruses from other PICTs, particularly PF. In addition introduction of South-east Asian genotype I viruses also contributed to the circulation of DENV-1 in NC, at least transiently in 2002–2003 [8,20] and sustainably in 2012–2013. The fact that introductions events led to sustainable circulation of DENV-1 and the occurrence of three consecutive outbreaks suggests that, at least until 2013, the NC population did not already had acquired sufficient herd immunity against DENV-1. The observation that NC experienced strong positive human migratory threshold during the past 5 years suggest that the pool of susceptible hosts may have been resupplied by new residents [22]. Another factor that could have contributed to maintain the pool of susceptible hosts is the limitation of DENV-1 infection rates during the 2008–2009 outbreak, due to transient cross-protective immunity provided by the co-circulation of DENV-4 [23]. An alternative factor that might have contributed to maintain the proportion of susceptible hosts to a level compatible with sustained DENV-1 transmission is the vector control pressure. For several years, NC Health Authorities have invested lots of efforts in maintaining efficient vector control. Combined to the NC standard of living (air conditioning…) and to climate conditions less favorable to DENV transmission during the cool season, vector control measures might have contributed to modify dengue epidemiological profile. An additional factor that could have contributed to sustainable transmission of DENV-1 in NC in 2012–2013 is the emergence of Asian genotype I. Indeed, this is the first evidence of a genotype switch (Pacific to Asian) in NC and in the Pacific region. This observation supports the hypothesis that transmission of Asian genotype I was particularly efficient in the context of NC in 2012–2013. Indeed it has been shown that genotype switch can favour the persistence of a serotype in a specific environment and that distinct genotypes can display different epidemic potential [6,7,24,25]. Finally, what has been observed in NC might be the beginning of a new situation regarding the dengue circulation profile in the Pacific.

## Competing interests

The authors declare that they have no competing interests.

## Authors’ contributions

MDR designed the experiments, performed the molecular experiments, analyzed the data and drafted the manuscript. VMCL conceived the study, designed the experiments and drafted the manuscript. MA designed the experiments, performed the molecular experiments and analyzed the data. OOC performed the molecular experiments and analyzed the data. CR analyzed the data. ACG and AG carried out the clinical diagnosis. JA, JPG and AP contributed to materials and data analysis and revised the manuscript. EN and SC critically revised the manuscript for intellectual content. All authors read and approved the final manuscript.

## Supplementary Material

Additional file 1 List of the PICTs DENV-1 strains analyzed in this study. Click here for file
