# Supplementary material for: Epidemiological and molecular features of dengue virus type-1 in New Caledonia, South Pacific, 2001–2013
Source: Virol J. 2014 Mar 31;11:61. doi: 10.1186/1743-422X-11-61 (PMC3997821; doi:10.1186/1743-422X-11-61)
Supplement: Additional file 1 — List of the PICTs DENV-1 strains analyzed in this study. [file 1743-422X-11-61-S1.pdf]

Additional File 1. List of the PICTs DENV-1 strains analysed in this study

ID	Country	Localisation	Sample	Collection date	GenBank	Reference
FJ12/JX298567	Fiji	_	_	2012	JX298567	Pyke, unpublished
FJ12/JX298568	Fiji	_	_	2012	JX298568	Pyke, unpublished
FJ11/JX298570	Fiji	_	_	2011	JX298570	Pyke, unpublished
FJ11/JX298571	Fiji	_	_	2011	JX298571	Northill and Pyke, unpublished
KB12/JX298575	Kiribati	_	_	2012	JX298575	Pyke, unpublished
NC13/090113-396	New Caledonia	Noumea	Serum	2013	KF764699	This study
NC13/120113-613	New Caledonia	La Foa	Serum	2013	KF764700	This study
NC13/100113-537	New Caledonia	Dumbea	Serum	2013	KJ399986	This study
NC13/170113-1031	New Caledonia	Mont Dore	Serum	2013	KJ399987	This study
NC13/210513-18438	New Caledonia	Dumbea	Serum	2013	KJ399988	This study
NC13/210513-18759	New Caledonia	Dumbea	Serum	2013	KJ399989	This study
NC13/270513-18965	New Caledonia	Noumea	Serum	2013	KJ399991	This study
NC13/020713-20466	New Caledonia	Paita	Serum	2013	KJ399990	This study
NC12/170212-262	New Caledonia	Ponerihouen	Cell culture	2012	KC316018	This study
NC12/170212-256	New Caledonia	Noumea	Cell culture	2012	KC316020	This study
NC12/170212-258 #	New Caledonia	Noumea	Cell culture	2012	KJ399992	This study
NC12/060312-435 #	New Caledonia	Noumea	Cell culture	2012	KJ399993	This study
NC12/070312-453	New Caledonia	Noumea	Serum	2012	KJ399994	This study
NC12/120312-489	New Caledonia	Noumea	Serum	2012	KC316016	This study
NC12/120312-496 #	New Caledonia	Ouegoa	Cell culture	2012	KC316021	This study
NC12/260312-593 #	New Caledonia	Noumea	Cell culture	2012	KJ399995	This study
NC12/100412-732 #	New Caledonia	Noumea	Cell culture	2012	KJ399996	This study
NC12/160412-791 #	New Caledonia	Noumea	Cell culture	2012	KC316022	This study
NC12/230412-873	New Caledonia	Tontouta	Cell culture	2012	KC316017	This study
NC12/270412-911	New Caledonia	Dumbea	Cell culture	2012	KC316014	This study
NC12/270412-920	New Caledonia	Mare	Cell culture	2012	KC316015	This study
NC12/300412-924 #	New Caledonia	Noumea	Cell culture	2012	KJ399997	This study
NC12/030512-948 #	New Caledonia	Mont Dore	Cell culture	2012	KJ399998	This study
NC12/110512-1087 #	New Caledonia	We	Cell culture	2012	KJ399999	This study
NC12/160512-1171 #	New Caledonia	Dumbea	Cell culture	2012	KJ400000	This study
NC12/270612-1875 #	New Caledonia	Noumea	Serum	2012	KJ400001	This study
NC12/230512-1283	New Caledonia	Pouembout	Cell culture	2012	KC316019	This study
NC12/231112-2877 #	New Caledonia	Dumbea	Cell culture	2012	KC854412	This study
NC12/181212-3578	New Caledonia	Dumbea	Serum	2012	KC854414	This study
NC12/181212-3574	New Caledonia	Kone	Serum	2012	KC854413	This study
NC12/191212-3601 #	New Caledonia	Lifou	Serum	2012	KC854415	This study
NC10/070610-960	New Caledonia	Noumea	Cell culture	2010	JQ655091	This study
NC10/150710-1096	New Caledonia	Mont-Dore	Cell culture	2010	JQ655092	This study
NC10/080810-1138	New Caledonia	Noumea	Cell culture	2010	JQ655093	This study
NC09/050109-33	New Caledonia	Noumea	Serum	2009	JQ655077	This study
NC09/240109-772	New Caledonia	Noumea	Serum	2009	JQ655078	This study
NC09/130209-2229	New Caledonia	Thio	Serum	2009	JQ655079	This study
NC09/160209-2235	New Caledonia	Noumea	Serum	2009	JQ655080	This study
NC09/250209-3584	New Caledonia	Noumea	Serum	2009	JQ655081	This study
NC09/300509-13639	New Caledonia	Noumea	Cell culture	2009	JQ655082	This study
NC09/270709-14311	New Caledonia	Poya	Serum	2009	JQ655083	This study
NC09/140809-14415	New Caledonia	Yate	Cell culture	2009	JQ655084	This study
NC08/280108-160	New Caledonia	Noumea	Serum	2008	JQ655069	This study
NC08/110208-284	New Caledonia	Dumbea	Serum	2008	JQ655070	This study
NC08/250208-468 *	New Caledonia	Noumea	Serum	2008	JQ655071	This study
NC08/912 °	New Caledonia	_	_	2008	_	Aaskov et al., unpublished

NC08/140308-1110 °	New Caledonia	Noumea	_	2008	_	Aaskov et al., unpublished
NC08/140708-3629	New Caledonia	Noumea	Serum	2008	JQ655072	This study
NC08/031008-4048 *	New Caledonia	Dumbea	Serum	2008	JQ655073	This study
NC08/011208-4575 °	New Caledonia	Yate	Serum	2008	JQ655074	This study
NC08/121208-4844 *	New Caledonia	Noumea	Serum	2008	JQ655075	This study
NC08/291208-5142 *	New Caledonia	Noumea	Serum	2008	JQ655076	This study
NC07/180407-286	New Caledonia	Noumea	Serum	2007	JQ655067	This study
NC07/030507-418	New Caledonia	Mont-Dore	Serum	2007	JQ655068	This study
NC07/746	New Caledonia	_	_	2007	_	Aaskov et al., unpublished
NC04/531	New Caledonia	_	_	2004	_	Aaskov et al., unpublished
NC04/050504-1477	New Caledonia	Noumea	Serum	2004	JQ655065	This study
NC04/1791	New Caledonia	Mont-Dore	_	2004	_	Aaskov et al., unpublished
NC03/501	New Caledonia	Noumea	_	2003	_	8
NC03/691	New Caledonia	Thio	_	2003	_	20
NC03/1606	New Caledonia	Lifou	_	2003	_	8
NC03/3260	New Caledonia	Ouvea	_	2003	_	20
NC03/5594	New Caledonia	Poindimie	_	2003	_	8
NC03/7246	New Caledonia	_	_	2003	_	20
NC03/020903-7773	New Caledonia	Noumea	Serum	2003	JQ655061	This study
NC02/38	New Caledonia	Noumea	_	2002	_	8
NC02/401	New Caledonia	_	_	2002	_	8
NC02/010702-739	New Caledonia	Noumea	Serum	2002	JQ655052	This study
NC02/765	New Caledonia	_	_	2002	_	20
NC02/882	New Caledonia	Koumac	_	2002	_	8
NC02/1061	New Caledonia	_	_	2002	_	Aaskov et al., unpublished
NC02/1143	New Caledonia	_	_	2002	_	Aaskov et al., unpublished
NC01/270601-515	New Caledonia	Noumea	Cell culture	2001	JQ655049	This study
NU12/JX298572	Niue	_	_	2012	JX298572	Pyke, unpublished
NU12/JX298573	Niue	_	_	2012	JX298573	Pyke, unpublished
PF09/180509-138	French Polynesia	_	Cell culture	2009	JQ655046	Aubry et al., unpublished
PF08/230608-174	French Polynesia	_	Cell culture	2008	JQ655032	Aubry et al., unpublished
PF07/030107-40	French Polynesia	_	Cell culture	2007	JQ654973	Aubry et al., unpublished
PF06/130306-52	French Polynesia	_	_	2006	_	9
PF05/290305-145	French Polynesia	_	_	2005	_	9
PF04/250304-183	French Polynesia	_	_	2004	_	9
PF03/060103-45	French Polynesia	_	_	2003	_	9
PF02/140102-10	French Polynesia	_	_	2002	_	9
PF01/050201-19	French Polynesia	_	_	2001	_	9
VU10/180210-358	Vanuatu	_	Serum	2010	JQ655096	This study

* E gene DENV-1 strain identical to NC08/110208-284
° E gene DENV-1 strain identical to NC08/280108-160
E gene DENV-1 strain identical to NC12/170212-256
